# A novel role of microRNA 17-5p in the modulation of circadian rhythm

**DOI:** 10.1038/srep30070

**Published:** 2016-07-21

**Authors:** Qian Gao, Lan Zhou, Su-Yu Yang, Ji-Min Cao

**Affiliations:** 1 Department of Physiology, Institute of Basic Medical Sciences, Chinese Academy of Medical Sciences, School of Basic Medicine, Peking Union Medical College, Beijing 100005, China

## Abstract

The circadian clock helps living organisms to adjust their physiology and behaviour to adapt environmental day-night cycles. The period length of circadian rhythm reflects the endogenous cycle transition rate and is modulated by environmental cues or internal molecules, and the latter are of substantial importance but remain poorly revealed. Here, we demonstrated that microRNA 17-5p (miR-17-5p), which has been associated with tumours, was an important factor in controlling the circadian period. MiR-17-5p was rhythmically expressed in synchronised fibroblasts and mouse master clock suprachiasmatic nuclei (SCN). MiR-17-5p and the gene *Clock* exhibited a reciprocal regulation: miR-17-5p inhibited the translation of *Clock* by targeting the 3′UTR (untranslated region) of *Clock* mRNA, whereas the CLOCK protein directly bound to the promoter of *miR-17* and enhanced its transcription and production of miR-17-5p. In addition, miR-17-5p suppressed the expression of *Npas2*. At the cellular level, bidirectional changes in miR-17-5p or CLOCK resulted in CRY1 elevation. Accordingly, *in vivo*, both increase and decrease of miR-17-5p in the mouse SCN led to an increase in CRY1 level and shortening of the free-running period. We conclude that miR-17-5p has an important role in the inspection and stabilisation of the circadian-clock period by interacting with *Clock* and *Npas2* and potentially via the output of CRY1.

The circadian rhythm, which comprises a period of approximately 24 hours, regulates many biological aspects, including physiology and behaviour, in nearly all organisms. The mammalian endogenous central circadian oscillator (circadian clock) is located in the hypothalamic suprachiasmatic nuclei (SCN)^1^. One molecular mechanism underlying circadian clock is the multiple-interacted transcription-translation feedback loops, in which many clock genes, including *Clock* (circadian locomotor output cycles kaput), *Bmal1* (brain and muscle ARNT-like protein 1), *Per1* (period 1) and *Cry1* (cryptochrome 1), are involved^2^. CLOCK and BMAL1, the two key positive regulators of the circadian clock, promote the transcription of *Per1*, *Per2*, *Per3*, *Cry1*, *Cry2* and many other genes^3^,^4^. The PER-CRY complex can translocate into the nucleus and inhibit the transcription of specific genes mediated by the CLOCK/BMAL1 heterodimer^5^,^6^. The molecular feedback loops are accurately modulated by a substantial number of molecules. In recent years, many other factors were described as part of the clock mechanism to explain how clock genes are regulated at the transcriptional, translational and post-translational levels, and the importance of posttranscriptional regulation has gradually been recognised. MicroRNAs (miRNAs, miR) comprise a set of molecules that have a potential ability to fine-tune biological timing processes.

MiRNAs are members of non-coding RNAs and comprise approximately 22-nucleotide single-stranded RNAs. In animals, miRNA biogenesis is initiated with the production of primary miRNAs (pri-miRNA), which are transcribed from miRNA genes or the introns of protein-coding genes. Pri-miRNAs undergo a series of processing steps, including the generation of a stem-looped structure referred to as pre-miRNA, the exportation of pre-miRNAs from the nucleus to the cytoplasm, and the formation of a duplex complex that contains single-stranded mature miRNAs. Mature miRNAs associate with components of the RNA-induced silencing complex (RISC) to mediate specific mRNA degradation or translational repression. MiRNAs recognise the 3′ untranslated region (3′UTR) of target messenger RNA (mRNA) via base-paring interactions to regulate mRNA stability or translation^7^. MiRNAs have been implicated in numerous processes, including brain development, neuronal differentiation and synaptic plasticity^8^,^9^,^10^. Many miRNAs have been reported to be involved in diseases, such as Alzheimer’s disease^11^.

Increasing evidences indicate that miRNAs play fairly important roles in the modulation of the mammalian circadian clock^12^. Cheng *et al*.^13^ reported that brain specific miR-219 and miR-132 promote circadian processes and attenuate the entraining effects of light, respectively. MiR-122, a highly abundant hepatocyte-specific miRNA, is under the circadian control and is linked to the circadian regulation of lipid metabolism and the rhythmic expression profile of the circadian deadenylase *Nocturnin* in mouse liver^14^,^15^. Some miRNAs have been identified to oscillate in the mouse retina^16^ or affect the dynamic regulation of the circadian clock in skeletal muscle^17^. Other reports have indicated that specific miRNAs could target clock genes at the molecular level. However, these studies did not determine the specific functions of the miRNAs relevant to the physiological rhythms^18^,^19^,^20^.

The present study aimed to identify miRNAs that target the clock genes and regulate the circadian locomotor rhythm. Based on the preliminary screening study, we specifically focused on the regulatory function of miRNA-17-5p in the circadian rhythm and the underlying molecular mechanisms in a mouse model.

## Results

### MiR-17-5p represses the translation of *Clock* by targeting its 3′UTR

We used three computational prediction algorithms, miRanda, PicTar and TargetScan, to screen miRNAs that target the 3′UTR of *Clock* mRNA. The miRNAs with a high score or rank in all the three prediction algorithms were candidates (Fig. S1A). We compiled the results and identified the top 10 candidate miRNAs to validate their functions via a dual luciferase reporter assay. Cultured 293T cells were transfected with candidate miRNA mimics and luciferase expression constructs that contained the murine *Clock* transcript 3′UTR (Fig. S1B). The candidate miRNA mimics were also introduced to NIH/3T3 cells for further validation of the precise influences of candidate miRNAs on the expression of *Clock*. Among these miRNAs, we focused on miR-17-5p because the influence of miR-17-5p transfection on *Clock* expression in NIH/3T3 cells (Fig. S1C) was similar with the dual luciferase reporter assay (Fig. S1B). MiR-17-5p significantly decreased the expression of *Clock* in both assays. Thus, we demonstrated that miR-17-5p inhibited *Clock* expression by targeting its 3′UTR.

**Figure 1:**
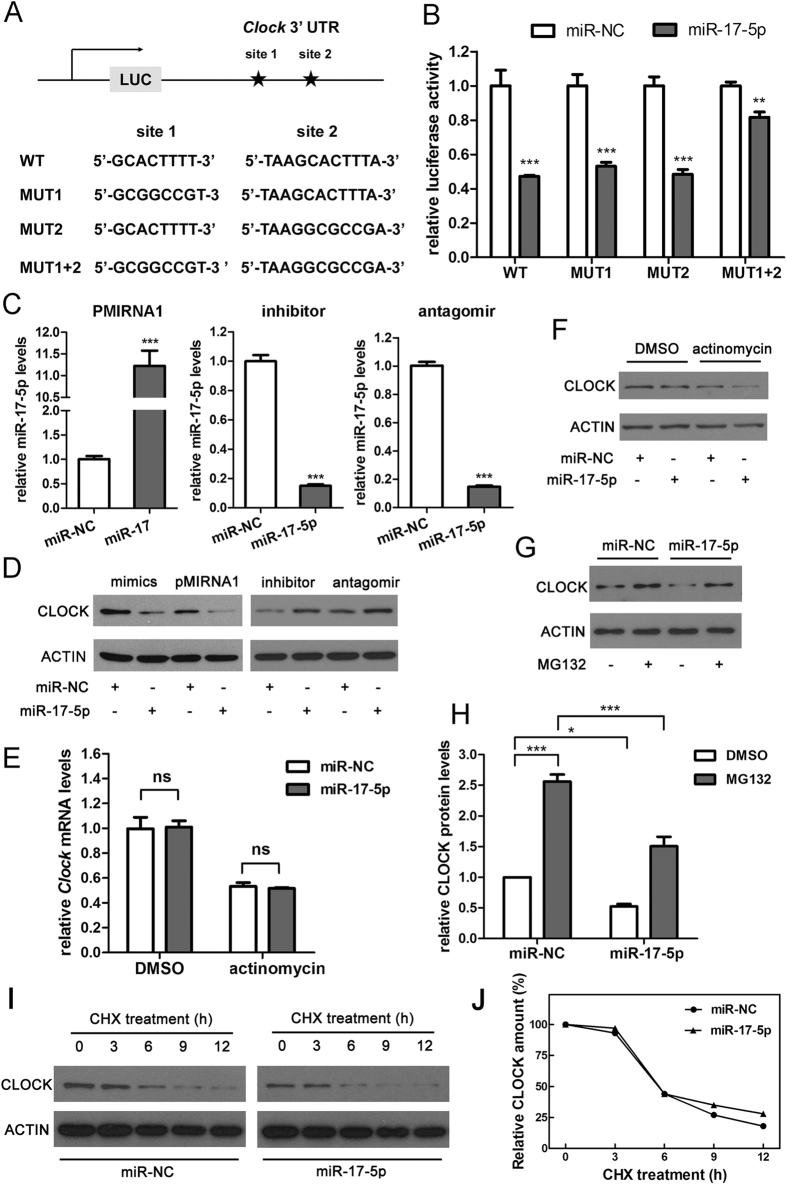
Identification of two major binding sites for miR-17-5p on the 3′UTR of *Clock* mRNA. (**A**) Diagram indicates two putative binding sites on the *Clock* 3′UTR. (**B**) Luciferase activities of the wild-type and mutant constructs of the *Clock* 3′UTR in 293T cells transfected with miR-NC or miR-17-5p. (**C**) RT-qPCR of miR-17-5p and (**D**) Western blots of CLOCK in N2a cells transfected with pMIRNA1 plasmid and mimics, inhibitor and antagomir of miR-17-5p. Nonsense sequences different from the miRNAs are used as a negative control (miR-NC) and set to 1.0 for RT-qPCR analyses. (**E,F**) RT-qPCR for *Clock* mRNA and Western blotting for CLOCK protein of N2a cells were preceded by incubation with or without 2 ng/μl actinomycin for 6 hours. (**G,H**) Western blot analysis of CLOCK in N2a cell lysates. Cells were transfected with miR-NC or miR-17-5p mimics followed by treatment with 50 nM MG-132 or DMSO for 8 hours. (**I, J**) Stability of endogenous CLOCK in the N2a cells transfected with miR-NC or miR-17-5p mimics and treated with 30 μg/ml CHX. CLOCK protein levels at 0 h were set to 100%. *P < 0.05, **P < 0.01, ***P < 0.001 *vs.* respective controls. N = 3 per group.

MiRNA has “seed sequences” that may bind to the 3′UTR of a target gene according to the Watson-Crick complementary principal. To confirm the interaction sites between miR-17-5p and the *Clock* 3′UTR, we identified two putative recognition sites in the *Clock* 3′UTR by utilising TargetScan. As shown in Fig. 1A, the first sequence was located at 1019–1025 (site 1) and the second was at 3841–3848 (site 2) of the *Clock* 3′UTR. We generated the point mutants of site 1, site 2 or both sites of the *Clock* 3′UTR. The double mutation of *Clock* 3′UTR significantly attenuated the inhibitory effect of miR-17-5p on *Luc* expression (Fig. 1B). As this attenuation did not reach 100%, we speculated that there might be other interacting regions on the *Clock* 3′UTR for miR-17-5p. Nevertheless, these two sites were primarily responsible for the binding and inhibition of miR-17-5p on *Clock* mRNA.

To further validate the impact of miR-17-5p on *Clock*, we performed overexpression and knockdown assays in cell lines. We transfected Neuro-2a (N2a) cells with miR-17-5p mimics or a pMIRNA1 overexpression construct with the insertion of pre-miR-17. The overexpression vector had two products, miR-17-5p and miR-17-3p. The transfection of the pMIRNA-miR-17 construct increased the abundance of mature miR-17-5p by more than 10 fold (Fig. 1C). Inhibitors and antagomirs of miRNAs comprise single-strand sequences complementary to a specific miRNA. They had the same sequence but with different modifications. We observed that the application of inhibitor or antagomir sharply repressed the endogenous expression of miR-17-5p (Fig. 1C). Western blot results indicated that the overexpression of miR-17-5p substantially decreased the level of CLOCK protein, whereas a miR-17-5p inhibitor or antagomir promoted it (Fig. 1D). These findings demonstrated that miR-17-5p inhibited the expression of *Clock*. In general, miRNAs inhibit gene expression by degrading mRNA or repressing protein translation. Our results revealed that blocking transcription via actinomycin significantly decreased the *Clock* mRNA level and introducing miR-17-5p into cells did not exert a further influence on the *Clock* mRNA (Fig. 1E). Moreover, miR-17-5p reduced the CLOCK protein level regardless of transcription blocking (Fig. 1F), suggesting that miR-17-5p may potentially affect the renewal of CLOCK protein, rather than the degradation of *Clock* mRNA.

To further clarify the action mechanism of miR-17-5p on *Clock*, we performed experiments at the translation process. As shown in Fig. 1G,H, when protein degradation was blocked by MG-132, an inhibitor of major proteasomes, miR-17-5p could still decrease the CLOCK protein level compared with the nonsense sequence. This finding suggests that miR-17-5p represses the translation of *Clock*. However, the possibility that miR-17-5p promotes the degradation of CLOCK cannot be excluded in this condition. Thus, we used cycloheximide (CHX), an inhibitor of protein synthesis in eukaryotic cells, to inhibit the synthesis of CLOCK and to determine whether miR-17-5p would affect the degradation speed of CLOCK. Assessment of the CLOCK protein half-life demonstrated that miR-17-5p did not affect the stability of CLOCK (Fig. 1I,J). These experiments suggest that miR-17-5p inhibits the translation, rather than promotes the mRNA degradation, of *Clock*.

### MiR-17-5p is rhythmically expressed in synchronised NIH/3T3 cells

**Figure 2:**
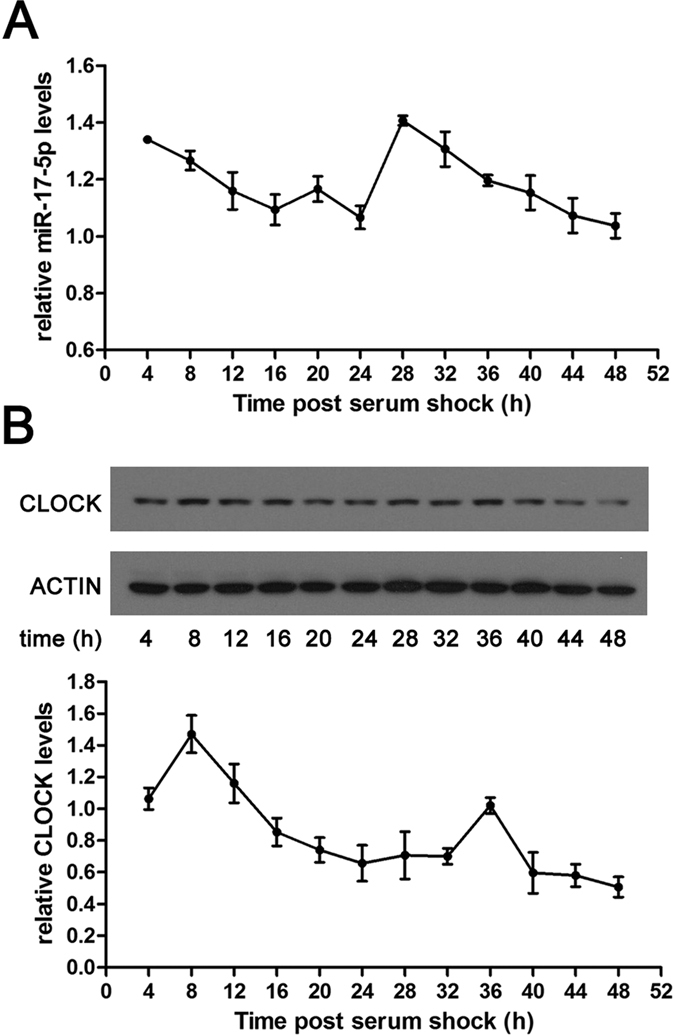
Rhythmic expression of CLOCK and miR-17-5p in synchronised NIH/3T3 cells. (**A,B**) Temporal expression patterns of miR-17-5p (**A**) or CLOCK protein (**B**) in NIH/3T3 cells following serum shock. Cells were synchronised at 0 h by 50% horse serum for 2 h and were subsequently cultured in 0.5% serum. Cells were collected every 4 h for two days followed by RT-qPCR or Western blotting. A representative example of Western blots (upper of B) and the grey values (lower of B) of each corresponding time point of three independent experiments are shown in panel B. Each n = 3 in A or B.

To investigate the expression patterns of miR-17-5p and CLOCK, we synchronised NIH/3T3 cells with 50% horse serum. We demonstrated that miR-17-5p was expressed in a circadian oscillatory pattern in the synchronised cells (Fig. 2A). Western blot results indicated that CLOCK was rhythmically expressed with a shifted peak value compared with miR-17-5p (Fig. 2B), whereas miR-17-5p and CLOCK had similar oscillatory periods of approximately 24 hours in expression. These findings suggest that miR-17-5p may be under the circadian control.

### MiR-17-5p expresses in the mouse SCN in a circadian manner

**Figure 3:**
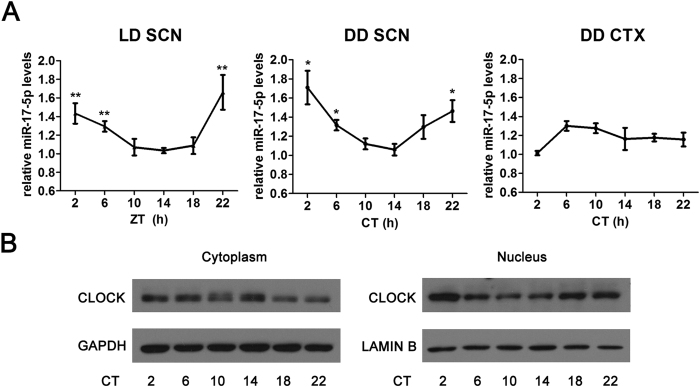
Rhythmic expression of miR-17-5p and CLOCK in the SCN of C57BL/6J mice. (**A**) RT-qPCR results indicate the circadian expression pattern of miR-17-5p in mouse SCN or cortex (CTX) in LD 12:12 (h) or DD conditions. All values were compared with CT14. *P < 0.05, **P < 0.01, n = 3 per time point. (**B**) Western blots of CLOCK in the cytoplasm and nucleus of SCN neurons at different time points during the day.

We examined the miR-17-5p levels in the SCN during a 24-h period in mice in LD (light:dark) 12:12 (h) or DD (constant dark) circumstances. As shown in Fig. 3A, the miR-17-5p expression exhibited similar rhythmic patterns in both LD and DD. The relative miR-17-5p levels were higher at late night and early morning but were lower in the middle of the day. The miR-17-5p level in the cortex of the mouse brain did not exhibit an obvious circadian pattern compared with that in the SCN (Fig. 3A). The CLOCK protein levels in the cytoplasmic and nuclear regions were both rhythmic but were in an opposite phase (Fig. 3B), representing a phenomenon of CLOCK shuffling between the cytoplasm and nucleus^21^. It is notable that the miR-17-5p level increased to a zenith value when the cytoplasmic CLOCK decreased to a nadir value. We speculated that there was a specific association between miR-17-5p and CLOCK.

### The transcription of miR-17 is directly regulated by CLOCK

**Figure 4:**
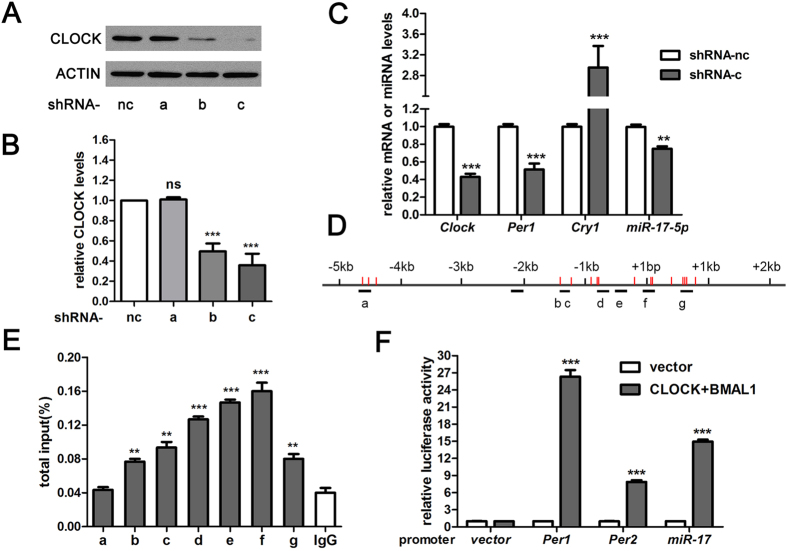
Direct regulation of miR-17-5p expression by CLOCK. (**A**) Western blots of CLOCK in N2a cells infected with three (*a*, *b* and *c*) different lentiviruses, which carried the interfering sequences that produced short-hairpin RNA that targeted *Clock* mRNA. (**B**) Quantitative analyses of A. Cells that expressed a nonsense interfering sequence served as a negative control (nc). (**C**) RT-qPCR quantified *Per1*, *Cry1*, *Clock* and miR-17-5p in N2a cells with an interference of *Clock*. (**D**) Schematic of the promoter region of *miR-17* from upstream 5 kb to downstream 2 kb scaled by black vertical lines. +1 indicated the start site of transcription. E-boxes are depicted as red vertical lines. The primers near the putative binding sites are shown as short bold horizontal lines below the sequence and are labelled *a* to *g*. (**E**) RT-qPCR of putative regions in the *miR-17* promoter bound by CLOCK in the ChIP assay. The negative control samples were immunoprecipitated with rabbit IgG. (**F**) Dual luciferase reporter gene assays for transactivation of CLOCK and BMAL1 on the *miR-17* promoter. Empty vectors were used as a negative control, and *mPer1-Luc* and *mPer2-Luc* were used as positive controls. **P < 0.01, ***P < 0.001 *vs.* respective control. N = 3 per group.

The rhythmic expression patterns of miR-17-5p in synchronised cells and SCN implicate that miR-17-5p expression may be under the control of circadian clock. To demonstrate this speculation, we generated stable *Clock*-knockdown N2a cell line using a lentivirus-vector-based short hairpin RNA (shRNA) approach. Of the three designed shRNA sequences (*a*, *b* and *c*), shRNA-c had the highest interfering efficacy, it greatly reduced the CLOCK protein level and the mRNA level of *Clock* decreased by more than 50% (Fig. 4A–C). We used the cell line stably expressing shRNA-c to determine the expression of specific clock genes. The expression levels of two important CLOCK-regulated genes, *Per1* and *Cry1*, were decreased and increased, respectively. The expression of miR-17-5p was decreased (Fig. 4C), suggesting that the level of miR-17-5p is under the control of *Clock*.

To further confirm whether CLOCK directly regulates *miR-17*, we performed a chromatin immunoprecipitation (ChIP) assay. The upstream 5-kb and downstream 2-kb sequences of *miR-17* transcription start site were examined for putative CLOCK-binding sites. As CLOCK binds to the E-box sequence to activate transcription, we used eight pairs of ChIP-qPCR primers covering the putative binding sites (Fig. 4D). As shown in Fig. 4E, CLOCK bound to the promoter region of *miR-17*, approximately from the upstream 1.5 kb to the transcription start site. In addition, we cloned the promoter region of *miR-17* and validated the transactivation of CLOCK and BMAL1 on *miR-17-Luc*. As indicated in Fig. 4F, compared to the vector control, co-expression of *Clock* and *Bmal1* promoted the transcription of *miR-17-Luc*.

Taken together, these results indicate that CLOCK binds to the promoter of *miR-17* and regulates the expression of miR-17-5p.

### Intracerebroventricular injection of antagomir-17-5p accelerates the circadian rhythm of mice

**Figure 5:**
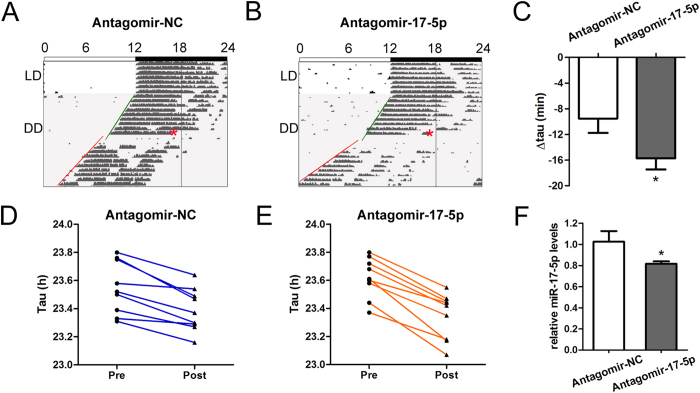
Acceleration of the free-running locomotor rhythms by antagomir-17-5p in mice. (**A,B**) Representative wheel-running actograms of C57BL/6J mice before and after *i.c.v.* injection of the negative control antagomir or miR-17-5p antagomir at CT20. The injection time point is indicated by a red asterisk. Activity onsets are indicated by green (before injection) or red (after injection) oblique lines. (**C**) Mean values of the delta free-running period (delta tau), which were −15.72 ± 1.72 min in the mice injected with agomir-17-5p and −9.51 ± 2.25 min in the control animals. *P = 0.0416 *vs.* scrambled, n = 9 per group. (**D,E**) Display of the free-running period of each mouse before and after *i.c.v.* injection of control antagomir (**D**) or miR-17-5p antagomir (**E**). (**F**) RT-qPCR analysis of miR-17-5p in the SCN following the injection of control antagomir or miR-17-5p antagomir. *P < 0.05, n = 3.

As shown above, miR-17-5p inhibited the expression of *Clock* and CLOCK promoted the transcription of miR-17-5p *in vitro*. Thus, we were interested in investigating the role of miR-17-5p in the circadian clock regulation *in vivo*. We performed an intracerebroventricular (*i.c.v.*) injection of antagomir-17-5p and investigated the effect of this antagomir on the circadian locomotor rhythms of mice, and observed a significant period length shortening after the injection of antagomir-17-5p compared with that produced by the control nonsense antagomir (Fig. 5A,B). The free-running period of the mice injected with antagomir-17-5p was shortened by approximately 17 min (Fig. 5C). The period lengths of the individual mice before and after the antagomir injection are shown in Fig. 5D,E. The antagomirs of miR-17-5p used in this study were similar with the inhibitors of miR-17-5p in action but with different modifications. Antagomirs comprise types of cholesterol-modified oligoribonucleotides complementary to miRNAs, they are more stable than inhibitors *in vivo* and thus are suitable for animal study. As reported, lateral ventricular injection is an efficient method for delivering tool drugs to the SCN of mice with minor injury to the brain. We selected CT20 as the injection time because this time point preceded the time of miR-17-5p expression peak. The injection of antagomir-17-5p contributed to the inhibition of miR-17-5p in the SCN of the mice even after 14 days (Fig. 5F). These findings indicate that miR-17-5p acts as a modulator of the circadian period length.

Because period shortening is associated with the output signalling of the circadian clock, we speculated that miR-17-5p may exert an effect on the output genes of the circadian clock, for example, *Per1* and *Pk2* (prokineticin 2). It has been shown that the expressions of the two genes reached their peak and valley at specific times with relevance to the cycles of circadian rhythm. Thus, the protein expression phases of the two genes are adjusted along with the change of period. When the free-running period is shortened, the phase would be different from the previous phase after multiple cycles. In accordance with these expectations, our findings indicated that the levels of PER1 and PK2 protein significantly decreased 14 days after the injection of antagomir-17-5p compared with the mice injected with the control (Fig. S2), in which the previous expression peaks (at the original CT12) were supposedly unchanged.

### Intracerebroventricular agomir-17-5p also promotes the circadian clock process in mice

As shown above, *i.c.v.* injection of antagomir-17-5p suppressed the expression/action of miR-17-5p in the SCN and led to an acceleration of the circadian locomotor rhythm and a shortening of the period length. We further examined whether agomir-17-5p administrated in the same way, a procedure supposed to increase the action of miR-17-5p in the SCN, would exert an opposite effect on the circadian period compared with antagomir-17-5p.

**Figure 6:**
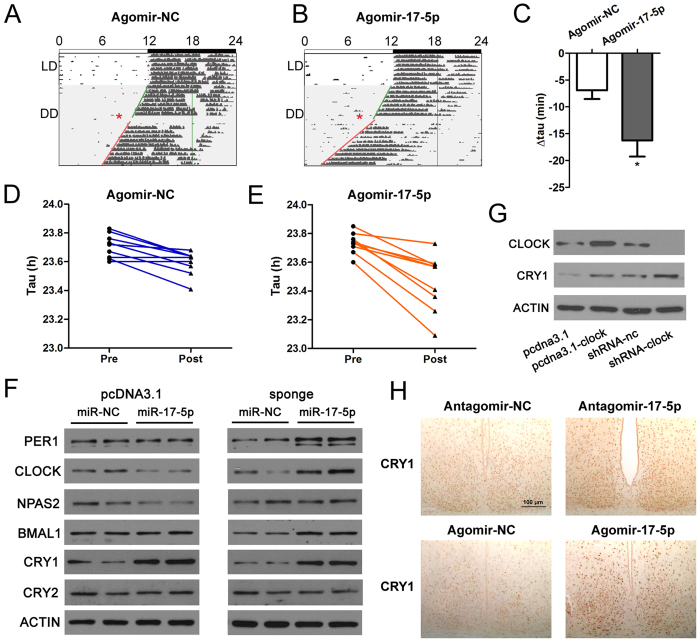
Agomir-17-5p also accelerated the free-running rhythm of mice. (**A**,**B**) Representative actograms of C57BL/6J mice that underwent *i.c.v.* injection of the control (agomir-NC) or miR-17-5p agomir (agomir-17-5p) at CT10. The activity onsets are depicted by coloured oblique lines, and the injection times are indicated by red asterisks. (**C**, **D**) Display of free-running periods of an individual mouse before and after the *i.c.v.* injection of control (**C**) or miR-17-5p agomir (**D**). (**E**) Mean values of delta free-running periods (delta tau), which were −16.27 ± 3.01 min in the mice injected with agomir-17-5p and −6.93 ± 1.63 min in the control animals. *P = 0.0150 *vs.* scrambled, n = 9 per group. (**F**) Western blotting indicates the effects of miR-17-5p on clock gene expression in N2a cells steadily transfected with control vector or pcDNA3.1-miR-17 (left) and control sponge or miR-17-5p sponge (right). Note that the overexpression or subtraction of miR-17-5p increased the CRY1 expression in N2a cells. (**G**) Western blotting indicates the overexpression and interference of *Clock* augmented CRY1 protein. (**H**) Immunochemical stains of CRY1 in the SCN of mice at CT12 underwent *i.c.v.* injections of miR-17-5p antagomirs or agomirs.

Agomirs comprise modified miRNAs and are similar with mimics in actions but are more stable and can be used *in vivo*. We delivered agomir-17-5p to the lateral ventricle of the mouse brains, a performance equal to an increase of miR-17-5p level in the SCN. We speculated that agomir-17-5p may exert an opposite action compared with the antagomir-17-5p. However, to our surprise, *i.c.v.* injection of agomir-17-5p also promoted the circadian clock process, as demonstrated by the shortening of the free-running period in mice. And, the period-shortening effect of agomir-17-5p was even greater than the antagomir-17-5p (Fig. 6A through 6E). The free-running periods of the individual mice before and after the injection of agomir-17-5p are shown in Fig. 6C,D. The free-running period of a mouse that received agomir-17-5p was shortened by even half an hour. These unexpected results suggest that there may be an equilibrium mechanism to stabilise the miR-17-5p level in the SCN, and changes in the miR-17-5p level would accelerate the circadian clock process and lead to period shortening, potentially via specific output signalling molecule(s).

We attempted to determine the mechanism(s) accounting for the puzzling phenomenon described above, i.e., why *i.c.v.* injection either of agomir-17-5p or of antagomir-17-5p shortened the period length. Plasmids for the overexpression of miR-17-5p were constructed and transfected to N2a cells. After screening for several generations with G418, a cell line stably overexpressing miR-17-5p was constructed. In addition, we packed the lentivirus that carried miR-17-5p sponges and infected N2a cells, aiming to use this tool to compete with the binding sites of the corresponding miRNAs and effectively attenuate the inhibitory effect of miR-17-5p on its target genes. The expression of several clock genes was assessed in these cell lines (Figs 6F and S3). The overexpression of miR-17-5p did not significantly affect the mRNA levels of most clock genes, with the exception of a slight increase in the mRNA levels of *Cry1*, *Cry2* and *Per2* (Fig. S3). The knockdown of miR-17-5p also did not affect the mRNA expression of most clock genes (Fig. S3). However, overexpression of miR-17-5p decreased the protein expression of CLOCK and its paralog, NPAS2 (Fig. 6F), whereas the inhibition of miR-17-5p increased the expression of CLOCK and several clock proteins, including BMAL1, PER1 and CRY1 (Fig. 6F).

As shown in Fig. 6F, both overexpression and knockdown of miR-17-5p increased CRY1 protein level. We are curious regarding how miR-17-5p affected the CRY1 protein level. CRY1 was not the direct target of miR-17-5p. Thus, we speculated that CLOCK may mediate the regulation of miR-17-5p on CRY1. In Fig. 6G, it was clear that both the overexpression and knockdown of *Clock* increased the CRY1 protein level, an evidence supporting the above speculation.

The increase of CRY1 protein level in response to bidirectional changes of miR-17-5p were also demonstrated *in vivo* (Fig. 6H). These results obtained at the cellular and integral levels were in accordance with the behavioural results that both increase and decrease of miR-17-5p in the SCN lead to an accelerated process of the circadian clock (period shortening). Taken together, these findings suggest that miR-17-5p is a distinct molecule in equilibrating the period length of the circadian rhythm, potentially via the modulation of CRY1 level through CLOCK in the SCN.

## Discussion

In recent years, miR-17-5p has gained attention not only for its potential roles in tumorigenesis^22^,^23^,^24^,^25^ and peripheral organ development^26^,^27^,^28^, but also for its roles in the development of the central nervous system (CNS)^29^. However, whether miR-17-5p plays a role in the regulation of circadian rhythm has not been reported. The present study demonstrated an interesting function of miR-17-5p in the regulation of circadian clock period.

One of the major findings of the present study was that miR-17-5p and *Clock* exhibited reciprocal regulation: miR-17-5p bound to two sites on the 3′UTR of *Clock* mRNA and strongly suppressed the expression of *Clock*, whereas CLOCK protein directly bound to the promoter of *miR-17* and enhanced the expression of miR-17-5p. *Clock* is the key gene that controls the circadian rhythm. Thus, these findings may basically endow miR-17-5p as a regulator of circadian rhythm. It’s notable that the 3′UTR of *Clock* mRNA (4556 bp) account for nearly half of the entire length of clock mRNA (9801 bp). According to the opinion of Stark *et al*.^30^, when confronting with miRNAs, genes are under selection to specifically avoid or take advantage of miRNA regulation. Thus, genes with longer lengths of 3′UTR may have more chances to be regulated by miRNAs. So both the absolute length and the relative length of the 3′UTR of *Clock* mRNA may likely provide abundant choices for post-translational regulation by miRNAs. We further identified that miR-17-5p decreased the CLOCK protein level not by promoting *Clock* mRNA or CLOCK protein degradation, but by repressing the translation of *Clock*.

In addition to the finding that miR-17-5p affected the CLOCK level in cell lines, we also demonstrated that miR-17-5p was rhythmically expressed in the SCN compared with its expression pattern in the cortex. This result further suggests a role of miR-17-5p in the function of the master clock. We determined that the oscillatory amplitude of the miR-17-5p level was not as large as *Per1* and other well-known clock genes, this result suggests a fine-tuning role of this miRNA in circadian clock regulation. Yang *et al*.^31^ demonstrated that circadian regulated miRNAs in *Drosophila*, including miR-263a and -263b, exhibited robust daily changes with moderate amplitudes. The moderate variation range of miRNAs may be the reason why they may fine-tune their target genes and the corresponding metabolic activities. We further demonstrated that miR-17-5p and CLOCK were expressed in different phases in synchronised cells and in opposite phases in the SCN. Moreover, miR-17-5p was under the control of CLOCK: CLOCK bound to the proximal promoter region of *miR-17* and promoted its transcription, and a stable decrease of CLOCK led to reduction of miR-17-5p. These experiments suggest that the circuit between miR-17-5p and CLOCK may comprise a mechanism that stabilises the molecular feedback loops of the circadian clock.

As shown in the present study, miR-17-5p was involved in the regulation of molecular clock. Thus, the miR-17-5p level may be relevant to the circadian clock in the SCN. It is reported that treatment with antagomir-17-5p abolished the growth of therapy-resistant neuroblastoma *in vivo*^32^. To confirm the importance of miR-17-5p in circadian rhythm regulation, we applied a specific antagomir *in vivo* to reduce the level of mature miR-17-5p in the mouse SCN. The decrease in miR-17-5p led to a shortening of the free-running period. It is possible that the inhibitory effect of miR-17-5p on the 3′UTR of *Clock* mRNA is relieved by antagomir-17-5p. According to the general concept, the shortening of the free-running period length in mice by antagomir-17-5p would prefigure a prolongation of the period length by agomir-17-5p. Intriguingly, the result was opposite to this speculation: *i.c.v.* injection of agomir-17-5p also shortened the free-running period length as antagomir-17-5p did, rather than prolonged it, and the period length-shortening effect of agomir-17-5p was even stronger than antagomir-17-5p. This phenomenon is unconventional to the general concept of biological regulation. We suggest that this bewildering regulatory algorithm may occur because one miRNA has several target genes, and the circadian clock circuit is orchestrated by complex feedback controls. Luo *et al*.^33^ also reported a similar phenomenon in an investigation of the role of miR-279 in the circadian control: they demonstrated that both overexpression and deletion of miR-279 disrupted the circadian rhythm in *Drosophila*. In the present study, miR-17-5p had two binding sites in the 3′UTR of *Clock* mRNA and functioned as a stronger inhibitor of *Clock* compared with its other targets. Thus, a smaller quantity of miR-17-5p may be sufficient to exert a moderate repression of CLOCK expression. The introduction of antagomir-17-5p weakened the inhibition of miR-17-5p on CLOCK expression, and thus promoted the circadian rhythm process. In addition, as indicated in Fig. 6F, the interference of miR-17-5p indirectly boosted the expression of several important clock genes on the protein level, which may potentially result in a circadian process impetus.

The injection of agomir-17-5p not only suppressed *Clock* expression but also affected additional genes. At the cellular level (shown in Figs S4 and 6F), miR-17-5p also targeted NPAS2, a paralog of CLOCK. Overexpression of miR-17-5p inhibited the expression of CLOCK and NPAS2, this effect may endow miR-17-5p the rationality to shorten the period length, as *Clock*- or *Npas2*-null mice display shortened free running period^34^.

We also focused on the potential output molecule(s) of the circadian clock by which miR-17-5p affected the period length, and demonstrated that a consistent increase or decrease of miR-17-5p lead to a unidirectional change of a clock gene, i.e., the promotion of *Cry1* expression, whereas *Clock* exhibited bidirectional changes in response to increase or decrease of miR-17-5p. This unusual regulatory context is worthy of further investigation. As already shown by other investigators, CLOCK and BMAL1 promote the transcription of *Per* and *Cry*. We therefore investigated the alterations in CRY1 expression following manipulation of the CLOCK levels in cell lines. The overexpression and knockdown of *Clock* increased the CRY1 levels. It is notable that the knockdown of *Clock* increased both the mRNA and protein levels of *Cry1* (Figs 4C and 6F). CRY1 may be an important output signaling molecule of *Clock* in mediating the period shortening elicited by miR-17-5p, although we cannot exclude other signaling molecules involved in the output process of rhythm.

The period length of circadian rhythm is affected by multiple factors at different tiers of regulation with complicated mechanisms which are not entirely clear at present. *Cry1* is only one probable explanation for the unconventional phenomenon described above, because we do not know why interfering *Clock* promotes the expression of CRY1 and how CRY1 shortens the free-running period. It is possible that *Clock* may reduce the expression of CRY1 through a unknown way when it promotes the transcription of E-box-containing genes. It has been reported that *Cry1* knockout leads to period shortening of the circadian locomotor rhythms in mice^35^. However, this observation does not mean a linear linkage between *Cry1* and period length. For example, *Cry1* transgenic mice had a similar period length as the wild-type mice^36^. Although the present study shows relevance between CRY1 and the period length, we do not intend to suggest a causal and exclusive output role of CRY1 in the regulation of circadian period by miR-17-5p. *Cry1* was not the only changeable gene in response to the increase or decrease of mir-17-5p and *Clock* in the present study. MiR-17-5p may target additional regulatory genes of the free-running period. These considerations may partially explain the contradictory changes of the circadian period in different experimental background, for example, the present study and the *Cry1* knockout study^35^.

In conclusion, the results of molecular, cellular and *in vivo* experiments demonstrated that the circadian-regulated miR-17-5p inhibits the translation of *Clock* by targeting the 3′UTR of *Clock* mRNA and participates in the regulation of free-running period in mice. In addition, *Npas2* takes a similar role as *Clock* in the function of miR-17-5p. MiR-17-5p may specifically act as an indicator and a stabiliser of the circadian-clock period, potentially via the CLOCK-mediated output of CRY1. Overall, miR-17-5p may be a novel factor in the modulation of the circadian molecular feedback loops and an intermediate molecule that provides circadian regulation to many other physiological and pathological pathways.

## Materials and Methods

### Cell culture, transfection and sample collection

Cell lines of 293T, NIH/3T3 and N2a were purchased from American Type Culture Collection (ATCC). The cells were cultured in Dulbecco’s modified Eagle’s medium (DMEM) that contained 10% foetal bovine serum (FBS), 100 units/ml penicillin and 100 mg/ml streptomycin at 37 °C and 5% CO_2_. The cells were transfected with plasmids or miRNA products (mimics or inhibitors) using Lipofectamine 2000 Transfection Reagent (Invitrogen, USA). For cell synchronisation assays, 1 × 10^6^ cells were seeded in a 6-cm petri dish and grew for 24 h. The cells were subsequently treated with 50% horse serum for 2 h followed by a replacement of DMEM with 0.5% FBS for the subsequent time. Samples were collected every 4 h after serum shock from 4 to 48 h.

### Dual luciferase reporter assay

The *Clock* 3′UTR (4.5 kb) and *Npas2* 3′UTR (1.2 kb) were amplified via PCR from the cDNA of the NIH/3T3 cell line and cloned to a pMIR-REPORT™ luciferase vector (Ambion, USA). The cell line 293T was seeded on a 24-well plate 24 h prior to transfection. MiRNA mimics, Clock 3′UTR luciferase vector and Renilla-luciferase vector (reference control) were transfected into 293T cells at the amounts of 20 pmol, 80 ng and 8 ng per well, respectively. Cell lysis and luciferase measurements were performed with a Dual-Luciferase Reporter Assay System (Promega, USA) according to the manufacturer’s instructions. The promoters of *miR-17* (1.8 kb), *Per1* (1.8 kb) and *Per2* (1.7 kb) were cloned from the genome of the NIH/3T3 cell line and ligated to pGL3-basic^37^. Constructs of 80 ng pGL3-basic, miR-17-Luc, Per1-Luc or Per2-Luc were mixed with 8 ng Renilla-luciferase vector, respectively, and transfected into 293T cells. Clock (700 ng) and Bmal1 (300 ng) were also introduced into the 293T cells. PcDNA3.1 was used as the control vector.

### Animals and brain tissue harvesting

Male C57BL/6J mice, age 8–10 weeks, were purchased from the Experimental Animal Center, Chinese Academy of Medical Sciences (Beijing). All animals were supplied with regular chow and water ad libitum and were housed in a standard animal maintenance facility under controlled light conditions. The mice were maintained in 12:12 (h) light:dark (LD) cycles for at least 14 days and were subsequently subjected to an environment of constant dark (DD). The mice were manipulated to perform voluntary running-wheel activities in both LD and DD conditions. The mice were euthanized, and the brains were harvested on the last day of LD or the second day of DD every 4 h for subsequent laboratory studies. The experiments in the dark phase were performed under red dim light (<5 lux). The animal use procedures were approved by the Life Ethics Committee of Peking Union Medical College and were conducted in compliance with the U.S. National Institutes of Health Guidelines for the Care and Use of Laboratory Animals (NIH Publication 85-23, revised 1985).

### RNA extraction and real-time quantitative PCR

The SCN tissues of the mice were excised from the brains. RNA extraction was performed with TRIzol reagent (Ambion, USA) according to the manufacturer’s instructions. The cDNA was generated, and real time quantitative PCR (RT-qPCR) was conducted on StepOne Plus (ABI, USA). The relative abundance of the transcripts was calculated by normalising to *mrpl19* (mitochondrial ribosomal protein L19) as an endogenous ref. 38. The specific RT-qPCR primers were listed in Table 1.

### Protein extraction and Western blotting

RIPA plus proteinase inhibitors were used to extract proteins from cells or SCN tissue samples. Nuclear/cytoplasm fractionation was performed using NE-PER nuclear and cytoplasmic extraction reagents (Thermo, USA). The cell or tissue lysates were normalised by measuring the protein concentration. The proteins were subsequently separated via SDS-PAGE and transferred to a nitrocellulose filter membrane for antibody recognition. The following antibodies were used: anti-CLOCK antibody (CST, USA), anti-NPAS2 antibody (Santa Cruz Biotechnology, USA), anti-CRY2 antibody (Santa Cruz Biotechnology, USA), Lamin B1 polyclonal antibody (ImmunoWay, USA), anti-β-actin rabbit polyclonal antibody (Santa Cruz Biotechnology, USA) and anti-GAPDH monoclonal antibody (MBL, Japan). The antibodies for PER1, CRY1 and BMAL1 were purchased from Abcam, UK. Quantity One software was used for the quantitative analyses of the protein expression.

### Plasmids and lentivirus infections

Constructs for Clock and Bmal1 were cloned from the cDNA of the mouse brains and ligated to pcDNA3.1. The sequences of pre-miR-17 and its adjacent region were cloned from the genome of the mice and ligated to pcDNA3.1 or pMIRNA1. The constructs were verified by sequencing. The plasmids were transiently or stably transfected to N2a cells as indicated. The N2a cells that expressed miR-17-5p were obtained by screening with 800 μg/ml G418. The interfering sequences that targeted the *Clock* mRNA are provided on the Sigma-Aldrich web site (http://www.sigmaaldrich.com/china-mainland/zh/life-science/functional-genomics-and-rnai/shrna/individual-genes.html). The sequences of the high scores were chosen for verification. Interfering sequence a was 5′-CTTCAGCAGTCAGTCCATAAA-3′, sequence b was 5′-CAGGACAGACAGATAAGATTT-3′ and sequence c was 5′-GAGAACATTCAGAGGTTTATA-3′. The oligonucleotides that contained the interfering sequences were linked to the pLKO.1 construct. The 293T cells were transfected with psPAX2, pMD2.G and pLKO.1, which carried different interfering sequences, respectively. After 48–72 h of transfection, the supernatant that contained mature virus was harvested and was used to infect the N2a cells. We screened the cells that stably expressed constructs via the addition of 2 μg/ml puromycin for several generations. MiR-17-5p that were sponge packed with adenovirus, a competitive inhibitor of small RNAs, were designed as reported^39^ and ordered from Hanbio Company (Shanghai, China).

### Monitoring of wheel-running activities and intracerebroventricular injection

The mice were individually housed in cages equipped with wireless running wheels. They were first entrained to LD 12:12 (h) for 14 days and subsequently transferred to DD for 8–9 days. The wheel-running activities were recorded by the VitalView programme (MiniMitter, USA), and the actograms of the wheel-running were analysed using ActiView software (MiniMitter, USA).

Intracerebroventricular (*i.c.v.*) injection of the agents into the lateral ventricle was performed according to the method we recently reported^40^. Briefly, the mouse was anesthetised with 20% urethane hydrate (1.3 g/kg, *i.p.*), and the head was fixed to a stereotaxic apparatus. The skull bone was exposed following a skin incision. A small hole (diameter 1 mm) was made perpendicularly to the skull with a skull drill. To create the hole, the coordinates (posterior, −0.46 mm from bregma; lateral, 1.0 mm from the midline; and dorsoventral, −2.3 mm from bregma) were used. The tip of a 24-gauge injector was inserted into the lateral ventricle following the hole. Injections of antagomirs (3 μl for each antagomir) were performed at CT20. Control experiments were performed with mice injected with scrambled antagomirs (each 3 μl) that had no complementarity to any known murine miRNAs. Following injection, the skull hole was rapidly sealed with dental cement, and the animal was transferred to the DD condition for free-running activity.

### Chromatin immunoprecipitation (ChIP)

N2a cells were cultured as above described. When the confluence reached approximately 95%, the cells were cross-linked with 1% formaldehyde, washed with PBS and transferred to an EP tube to prepare for lysis. Following nuclear protein extraction, the DNA was sheared to fragments of 200*−*1000 bp by sonication. A ChIP assay of the CLOCK-DNA complexes was performed with the CLOCK antibody. Purified DNA was subjected to real-time PCR. The detailed steps were processed with an EpiQuikTM Chromatin Immunoprecipitation Kit (Epigentek, USA) according to the manufacturer’s instructions.

### Immunohistochemistry

The whole brains of the mice were rapidly harvested at the indicated time point and fixed in 4% fresh paraformaldehyde for 48 h. The brain tissues were dehydrated in gradient ethanol and embedded in paraffin. SCN sections were cut, processed and immunostained for CLOCK, PER1 and PK2 using the following antibodies: rabbit polyclonal antibody against CRY1 (1:600, Abcam, USA), rabbit polyclonal antibody against PK2 (1:600, Abcam, UK) and rabbit monoclonal antibody against PER1 (1:600, Abcam, UK). Bright-field microscopy images were captured using a digital camera installed on a Leica microscope.

### Statistical analysis

The data are presented as the mean ± standard error of the mean (SEM). Statistical analyses were performed using GraphPad Prism software. The two-tailed t-test was used to compare the mean values of the two groups. The criterion for statistical significance was P < 0.05.

## Additional Information

**How to cite this article**: Gao, Q. *et al*. A novel role of microRNA 17-5p in the modulation of circadian rhythm. *Sci. Rep.* **6**, 30070; doi: 10.1038/srep30070 (2016).

## Supplementary Material

Supplementary Information

## Figures and Tables

**Table 1:** Primer sequences for RT-qPCR.

Gene	Forward primer	Reverse primer
*Mrpl19*	5′-CGCAAGCCTGTGACTGTCCATT-3′	5′-GGCAGTACCCTTCCTCTTCCCTAT-3′
*Clock*	5′-CCTATCCTACCTTCGCCACACA-3′	5′-TCCCGTGGAGCAACCTAGAT-3′
*Per1*	5′-TTGGCAGGCTTCGTGGACTTG-3′	5′-GCGGGAACGCTTTGCTTTAGAT-3′
*miR-17-5p*	5′-TCAGGTCAAAGTGCTTACAGTGC-3′	5′-GTGCAGGGTCCGAGGT-3′
*Cry1*	5′-ATCCACCATTTAGCCAGACACG-3′	5′-GACAGCCACATCCAACTTCCAG-3′
*Npas2*	5′-CCCACTACTACATCACCTACCACC-3′	5′-CTGTCTCCTTTCCACTCGAACATC-3′
*Bmal1*	5′-CCTTCCCGCAGCTAACAGC-3′	5′-CTCCGCGATCATTCGACCTAT-3′
*Per2*	5′-GACAGCAGCTTCTGGTCTGGACT-3′	5′-TTCTGAGTGTCTGAGGGCTCGTT-3′
*Cry2*	5′-GGGGACTCTGTCTATTGGCATCT-3′	5′-CTGGCTCTTGGGTAGGCATCT-3′

## References

[b1] Dunlap J. C. Molecular bases for circadian clocks. Cell 96, 271–290 (1999). 9988221 10.1016/s0092-8674(00)80566-8

[b2] Hastings M. H., Reddy A. B. & Maywood E. S. A clockwork web: circadian timing in brain and periphery, in health and disease. Nat Rev Neurosci 4, 649–661 (2003). 12894240 10.1038/nrn1177

[b3] Bunger M. K. . Mop3 is an essential component of the master circadian pacemaker in mammals. Cell 103, 1009–1017 (2000). 11163178 10.1016/s0092-8674(00)00205-1 PMC3779439

[b4] Gekakis N. . Role of the CLOCK protein in the mammalian circadian mechanism. Science 280, 1564–1569 (1998). 9616112 10.1126/science.280.5369.1564

[b5] Sangoram A. M. . Mammalian circadian autoregulatory loop: a timeless ortholog and mPer1 interact and negatively regulate CLOCK-BMAL1-induced transcription. Neuron 21, 1101–1113 (1998). 9856465 10.1016/s0896-6273(00)80627-3

[b6] Kume K. . mCRY1 and mCRY2 are essential components of the negative limb of the circadian clock feedback loop. Cell 98, 193–205 (1999). 10428031 10.1016/s0092-8674(00)81014-4

[b7] He L. & Hannon G. J. MicroRNAs: small RNAs with a big role in gene regulation. Nat Rev Genet 5, 522–531 (2004). 15211354 10.1038/nrg1379

[b8] Harraz M. M., Xu J. C., Guiberson N., Dawson T. M. & Dawson V. L. MiR-223 regulates the differentiation of immature neurons. Mol Cell Ther 2, 18 (2014). 25400937 10.1186/2052-8426-2-18 PMC4229944

[b9] Petri R., Malmevik J., Fasching L., Akerblom M. & Jakobsson J. miRNAs in brain development. Exp Cell Res 321, 84–89 (2014). 24099990 10.1016/j.yexcr.2013.09.022

[b10] Yoo A. S., Staahl B. T., Chen L. & Crabtree G. R. MicroRNA-mediated switching of chromatin-remodelling complexes in neural development. Nature 460, 642–646 (2009). 19561591 10.1038/nature08139 PMC2921580

[b11] Lukiw W. J., Zhao Y. & Cui J. G. An NF-kappaB-sensitive micro RNA-146a-mediated inflammatory circuit in Alzheimer disease and in stressed human brain cells. J Biol Chem 283, 31315–31322 (2008). 18801740 10.1074/jbc.M805371200 PMC2581572

[b12] Kojima S., Shingle D. L. & Green C. B. Post-transcriptional control of circadian rhythms. J Cell Sci 124, 311–320 (2011). 21242310 10.1242/jcs.065771 PMC3021995

[b13] Cheng H. Y. . microRNA modulation of circadian-clock period and entrainment. Neuron 54, 813–829 (2007). 17553428 10.1016/j.neuron.2007.05.017 PMC2590749

[b14] Gatfield D. . Integration of microRNA miR-122 in hepatic circadian gene expression. Genes Dev 23, 1313–1326 (2009). 19487572 10.1101/gad.1781009 PMC2701584

[b15] Kojima S., Gatfield D., Esau C. C. & Green C. B. MicroRNA-122 modulates the rhythmic expression profile of the circadian deadenylase Nocturnin in mouse liver. PLoS One 5, e11264 (2010). 20582318 10.1371/journal.pone.0011264 PMC2889834

[b16] Xu S., Witmer P. D., Lumayag S., Kovacs B. & Valle D. MicroRNA (miRNA) transcriptome of mouse retina and identification of a sensory organ-specific miRNA cluster. J Biol Chem 282, 25053–25066 (2007). 17597072 10.1074/jbc.M700501200

[b17] Zhou W., Li Y., Wang X., Wu L. & Wang Y. MiR-206-mediated dynamic mechanism of the mammalian circadian clock. BMC Syst Biol 5, 141 (2011). 21902842 10.1186/1752-0509-5-141 PMC3201034

[b18] Nagel R., Clijsters L. & Agami R. The miRNA-192/194 cluster regulates the Period gene family and the circadian clock. FEBS J 276, 5447–5455 (2009). 19682069 10.1111/j.1742-4658.2009.07229.x

[b19] Tan X. . Clock-controlled mir-142-3p can target its activator, Bmal1. BMC Mol Biol 13, 27 (2012). 22958478 10.1186/1471-2199-13-27 PMC3482555

[b20] Shende V. R., Neuendorff N. & Earnest D. J. Role of miR-142-3p in the post-transcriptional regulation of the clock gene Bmal1 in the mouse SCN. PLoS One 8, e65300 (2013). 23755214 10.1371/journal.pone.0065300 PMC3673942

[b21] Kondratov R. V. . BMAL1-dependent circadian oscillation of nuclear CLOCK: posttranslational events induced by dimerization of transcriptional activators of the mammalian clock system. Genes Dev 17, 1921–1932 (2003). 12897057 10.1101/gad.1099503 PMC196247

[b22] He L. . A microRNA polycistron as a potential human oncogene. Nature 435, 828–833 (2005). 15944707 10.1038/nature03552 PMC4599349

[b23] O’Donnell K. A., Wentzel E. A., Zeller K. I., Dang C. V. & Mendell J. T. c-Myc-regulated microRNAs modulate E2F1 expression. Nature 435, 839–843 (2005). 15944709 10.1038/nature03677

[b24] Hayashita Y. . A polycistronic microRNA cluster, miR-17-92, is overexpressed in human lung cancers and enhances cell proliferation. Cancer Res 65, 9628–9632 (2005). 16266980 10.1158/0008-5472.CAN-05-2352

[b25] Uziel T. . The miR-17~92 cluster collaborates with the Sonic Hedgehog pathway in medulloblastoma. Proc Natl Acad Sci USA 106, 2812–2817 (2009). 19196975 10.1073/pnas.0809579106 PMC2636735

[b26] Ventura A. . Targeted deletion reveals essential and overlapping functions of the miR-17 through 92 family of miRNA clusters. Cell 132, 875–886 (2008). 18329372 10.1016/j.cell.2008.02.019 PMC2323338

[b27] Zhang M. . Both miR-17-5p and miR-20a alleviate suppressive potential of myeloid-derived suppressor cells by modulating STAT3 expression. J Immunol 186, 4716–4724 (2011). 21383238 10.4049/jimmunol.1002989

[b28] Danielson L. S. . Cardiovascular dysregulation of miR-17-92 causes a lethal hypertrophic cardiomyopathy and arrhythmogenesis. FASEB J 27, 1460–1467 (2013). 23271053 10.1096/fj.12-221994 PMC3606524

[b29] Zhang Y. . The MicroRNA-17-92 cluster enhances axonal outgrowth in embryonic cortical neurons. J Neurosci 33, 6885–6894 (2013). 23595747 10.1523/JNEUROSCI.5180-12.2013 PMC3657758

[b30] Stark A., Brennecke J., Bushati N., Russell R. B. & Cohen S. M. Animal MicroRNAs confer robustness to gene expression and have a significant impact on 3′UTR evolution. Cell 123, 1133–1146 (2005). 16337999 10.1016/j.cell.2005.11.023

[b31] Yang M., Lee J. E., Padgett R. W. & Edery I. Circadian regulation of a limited set of conserved microRNAs in Drosophila. BMC Genomics 9, 83 (2008). 18284684 10.1186/1471-2164-9-83 PMC2263044

[b32] Fontana L. . Antagomir-17-5p abolishes the growth of therapy-resistant neuroblastoma through p21 and BIM. PLoS One 3, e2236 (2008). 18493594 10.1371/journal.pone.0002236 PMC2375057

[b33] Luo W. & Sehgal A. Regulation of circadian behavioral output via a MicroRNA-JAK/STAT circuit. Cell 148, 765–779 (2012). 22305007 10.1016/j.cell.2011.12.024 PMC3307393

[b34] DeBruyne J. P., Weaver D. R. & Reppert S. M. CLOCK and NPAS2 have overlapping roles in the suprachiasmatic circadian clock. Nat Neurosci 10, 543–545 (2007). 17417633 10.1038/nn1884 PMC2782643

[b35] van der Horst G. T. . Mammalian Cry1 and Cry2 are essential for maintenance of circadian rhythms. Nature 398, 627–630 (1999). 10217146 10.1038/19323

[b36] Okano S., Akashi M., Hayasaka K. & Nakajima O. Unusual circadian locomotor activity and pathophysiology in mutant CRY1 transgenic mice. Neurosci Lett 451, 246–251 (2009). 19159659 10.1016/j.neulet.2009.01.014

[b37] Hida A. . The human and mouse Period1 genes: five well-conserved E-boxes additively contribute to the enhancement of mPer1 transcription. Genomics 65, 224–233 (2000). 10857746 10.1006/geno.2000.6166

[b38] Chang H. C. & Guarente L. SIRT1 mediates central circadian control in the SCN by a mechanism that decays with aging. Cell 153, 1448–1460 (2013). 23791176 10.1016/j.cell.2013.05.027 PMC3748806

[b39] Ebert M. S., Neilson J. R. & Sharp P. A. MicroRNA sponges: competitive inhibitors of small RNAs in mammalian cells. Nat Methods 4, 721–726 (2007). 17694064 10.1038/nmeth1079 PMC3857099

[b40] Zhou L. . Activation of growth hormone secretagogue receptor induces time-dependent clock phase delay in mice. Am J Physiol Endocrinol Metab 307, E515–E526 (2014). 25074983 10.1152/ajpendo.00535.2013

