# Supplementary material for: A novel role of microRNA 17-5p in the modulation of circadian rhythm
Source: Sci Rep. 2016 Jul 21;6:30070. doi: 10.1038/srep30070 (PMC4954982; doi:10.1038/srep30070)
Supplement: Supplementary Information [file srep30070-s1.pdf]

A novel role of microRNA 17-5p in the modulation of circadian rhythm

Qian Gao, Lan Zhou, Su-Yu Yang, and Ji-Min Cao*

From the Department of Physiology, Institute of Basic Medical Sciences, Chinese Academy of Medical Sciences, School of Basic Medicine, Peking Union Medical College, Beijing 100005, China

* Corresponding author (email: caojimin@126.com. Tel: +86 10 6915 6959)

Supplementary information

Supplementary figures and legends

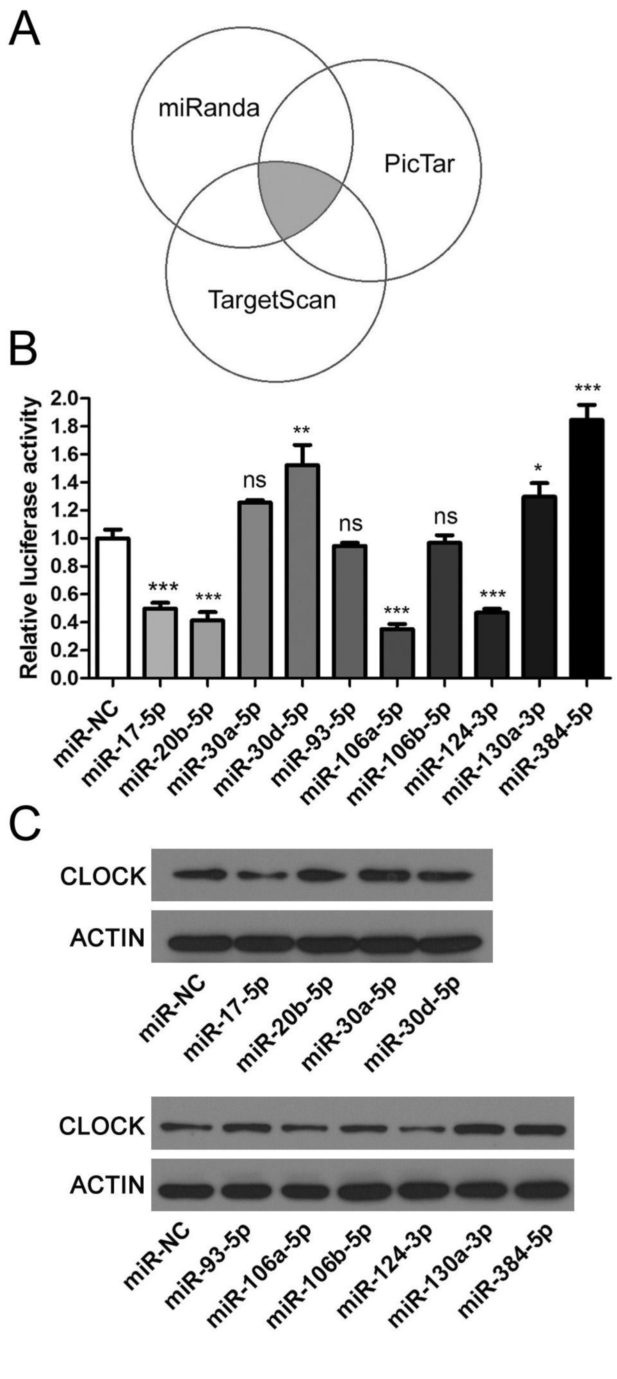

Figure S1. Screening for miRNAs targeting *clock* 3'UTR. (A) A schematic draw that illustrates the screening of miRNAs targeting *Clock* 3'UTR from three online softwares, miRanda, PicTar

and TargetScan. The grey area showed the common miRNAs in the three algorithms, and the miRNAs in this area were considered as candidates. (B) Effects of the candidate miRNA mimics on the *Clock* 3'UTR reporter. The 293T cells were transfected with luciferase reporter gene carrying *Clock* 3'UTR and miRNA mimics. The firefly relative luciferase activity was normalized with internal control, the sea pansy luciferase. Each group was compared with the nonsense mimics (negative control, miR-NC). * $P < 0.05$, ** $P < 0.01$, *** $P < 0.001$, n = 3 for each miRNA. (C) Western blots of the abundance of CLOCK protein after transfecting with miRNA mimics in NIH/3T3 cells. Beta-actin was used as loading control.

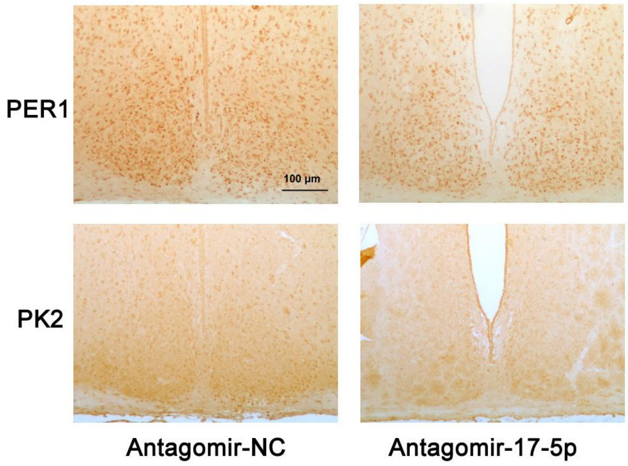

Figure S2. Immunohistochemical stains showing the effects of antagomir-17-5p *i.c.v.* injection on the protein expressions of circadian clock output genes *Per1* and *Pk2* in mice SCN. The SCN tissue harvesting time for both group (antagomir-nc and antagomir-17-5p) was at the CT12 of antagomir-nc group fourteen days after *i.c.v.* injection. Note that in the antagomir-17-5p treated mice, PER1 and PK2 were obviously lower than that of antagomir-NC treated mice in which the expression peaks of PER1 and PK2 were still at previous CT12, while the peaks of PER1 and PK2 in the antagomir-17-5p treated mice shifted and therefore showed lower levels. The decreasing of PER1 and PK2 checked at previous CT12 fourteen days after antagomir-17-5p injection was most probably due to period change of PER1 and PK2. The SCN was beside the third ventricle upon the optic chiasmata and was supposed to be easily affected by the antagomir-17-5p administrated via *i.c.v.* injection.

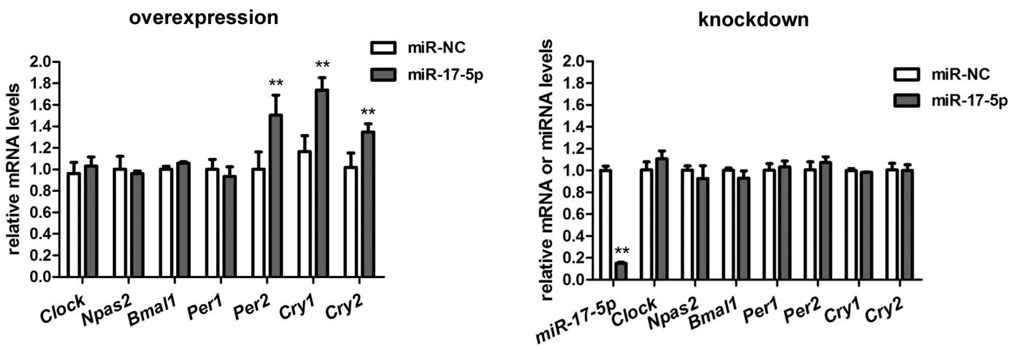

Figure S3. Changes of the mRNA levels for some clock genes in response to overexpression or knockdown of miR-17-5p in N2a cells assayed RT-qPCR. ** $P < 0.01$, *** $P < 0.001$ *vs.* miR-NC. N = 3 in each group.

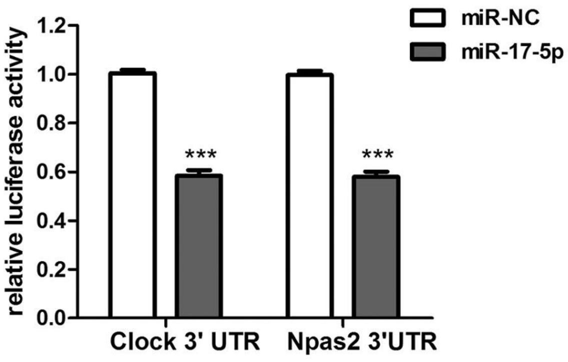

Figure S4. MiR-17-5p also targets the 3’UTR of *Npas2* mRNA. Effects of the miRNA mimics on the *Clock* and *Npas*2 3’UTR reporter. The 293T cells were transfected with luciferase reporter gene carrying respective 3’UTR and miRNA mimics. The firefly relative luciferase activity was normalized with internal control, the sea pansy luciferase. Each group was compared with the nonsense mimics (negative control, miR-NC). *** $P < 0.001$, N = 3 for each group.
